# Supplementary material for: Stress symptoms and positive coping during coronavirus disease 2019: The need to look at health from a gender perspective
Source: PLoS One. 2023 Jul 7;18(7):e0287055. doi: 10.1371/journal.pone.0287055 (PMC10328341; doi:10.1371/journal.pone.0287055)
Supplement: Attachment — Submitted filename: Response to the reviewers.docx [file pone.0287055.s001.docx]

Diana Marcela Walteros Acero, M.D.

Academic Editor

PLOS ONE

Regarding the manuscript entitled " Stress symptoms and positive coping during coronavirus disease 2019: the need to look at health from a gender perspective, that we submitted to the PLOS ONE, I take this opportunity to thank the reviewers for their comments and suggestions.

Comments from the Editor:

The context of the pandemic in that locality during the study period and the conditions related to the infection that could support the conclusions, for example, severity, disability, are unknown. The conclusions must consider the factors related to the infection and not only the comorbidities and it would be interesting to see if the disease had an effect on coping.

R= We explain the context of the epidemic in the state of Veracruz in the following text:

*In the case of the state of Veracruz, diabetes occurs as the 2nd. most prevalent disease, and cardiovascular diseases are the second cause of death (27-28), and the city of Veracruz, were the data were collected, was the city with more Covid-19 reported cases.*

*The city of Veracruz was one of the cities with the highest number of cases during the first months after the declaration of a pandemic by COVID-19 and it remained at a red epidemiological light for longer than most of the municipalities in the state of Veracruz. It is important to point out that the city of Veracruz also had a greater number of hospitals with diagnostic capacity for SARS CoV-2, but given that the number of confirmed cases was always high, the population that had the possibility of performing the diagnostic test in another site, looked for alternatives, such as going to the CESS, hence our sampling was not random, but for convenience.*

2. The network analysis was done taking into account the scores of the subscales, however it I s not clear how these aspects determine health and well-being with a gender perspective. It could be useful to do the network analysis with demographic variables and comorbidities. There is no description of biases or how to control them.

*R=Although the network analysis considers the factors that make up the two subscales of the positive coping instrument separately, the factors show more clearly how participants can cope with difficult situations, or fully enjoy positive situations.*

3. In the introduction, the gender perspective is blurred from the pandemic and the specific situation related to acute respiratory infection by SARS-CoV-2, it is raised from health in general, which makes it difficult to understand the working hypothesis.

*R=We have reduced the text that refers to the pandemic, so as not to divert attention from the gender perspective and how it should be considered in health.*

4. It remains to describe the municipality and the covid situation in the area at the project development site, which are contextual elements that can contribute to the analysis and generation of conclusions.

*R=In the case of the state of Veracruz, diabetes occurs as the 2nd. most prevalent disease, and cardiovascular diseases are the second cause of death (27-28), and the city of Veracruz, where the data were collected, was the city with more Covid-19 reported cases.*

5. The variables that are going to be used to analyze the gender perspective are not described, there is only sex. It is not clear if the PCWLS scale was validated in the sample studied.

*R= The PCWLS scale was validated by by Góngora-Coronado and Vázquez-Velázquez*.

6. Among the results, they affirm that differences were found in risk factors for contracting covid, it must be taken into account that this type of study does not allow us to rigorously identify these differences in risk factors, what is proposed is the analysis of the comorbidities of the people positive for Covid-19.

*R=According to the purpose of the study, we analyzed which aspects of positive coping are linked to the stress response, and with the presence/absence of important metabolic diseases in Mexico and in the context of the COVID-19 pandemic, from a gender perspective.*

7. It remains to be clarified whether it was possible to identify the analysis of the aspects that determine health and well-being from a gender perspective in the methods and present it in the conclusions in people with confirmed Covid-19.

*R=According to the purpose of the study, we analyzed which aspects of positive coping are linked to the stress response, and with the presence/absence of important metabolic diseases in Mexico and in the context of the COVID-19 pandemic, from a gender perspective. Sadly we do not have access to the data for the COVID confirmed data.*

The selection bias that occurs when selecting people who consult a health service for symptoms of Covid-19 must be declared, since they may have different conditions or characteristics from those who do not consult.

*R= The data was collected from those people who requested a diagnostic test for COVID-19. Therefore, the possible deviation could be in the fact that only those who arrived were sampled and not those who, having symptoms that suggested COVID.19, did not request the diagnosis.*

Journal Requirements:

When submitting your revision, we need you to address these additional requirements.

1. Please ensure that your manuscript meets PLOS ONE's style requirements, including those for file naming. The PLOS ONE style templates can be found at

R= *The format was checked up*

2. Please provide additional details regarding participant consent. In the ethics statement in the Methods and online submission information, please ensure that you have specified what type you obtained (for instance, written or verbal, and if verbal, how it was documented and witnessed). If your study included minors, state whether you obtained consent from parents or guardians. If the need for consent was waived by the ethics committee, please include this information.

*R=The procedures has been described in the Methods section.*

*Following all sanitary measures indicated during the COVID-19 pandemic, participants were verbally informed about the objectives of the protocol; once they agreed to participate, a Google Forms link via What’s App, was sent to them, to obtain informed consent via on line, and answer the general data questionnaire and the psychological tests. Personal data was processed in accordance with the policy of the Institute for Transparency, Access to Public Information, Protection of Personal Data. Once registered, a Google Forms link was sent through WhatsApp to answer a file with general data and psychological tests. This work was carried out following the Code of Ethics of the Declaration of Helsinki and approved by the Committee of Ethical Research of the CESS.*

3. Thank you for stating the following in the Acknowledgments Section of your manuscript:

"This research has been funded by Dirección General de Investigaciones of Universidad Santiago de Cali under call No. 01-2022."

We note that you have provided funding information that is not currently declared in your Funding Statement. However, funding information should not appear in the Acknowledgments section or other areas of your manuscript. We will only publish funding information present in the Funding Statement section of the online submission form.

Please remove any funding-related text from the manuscript and let us know how you would like to update your Funding Statement. Currently, your Funding Statement reads as follows:

"This research has been funded by Dirección General de Investigaciones of Universidad Santiago de Cali under call No. 01-2022. The funders had no role in study design, data collection and analysis, decision to publish, or preparation of the manuscript."

Please include your amended statements within your cover letter; we will change the online submission form on your behalf.

*R= The text has been modified and removed.*

4. We note that you have stated that you will provide repository information for your data at acceptance. Should your manuscript be accepted for publication, we will hold it until you provide the relevant accession numbers or DOIs necessary to access your data. If you wish to make changes to your Data Availability statement, please describe these changes in your cover letter and we will update your Data Availability statement to reflect the information you provide.

*R=The repository files have been included.*

5. Please include captions for your Supporting Information files at the end of your manuscript, and update any in-text citations to match accordingly. Please see our Supporting Information guidelines for more information: http://journals.plos.org/plosone/s/supporting-information.

All were incorporated according to the guide for the author.

S1_fig

S2_fig

S3_fig

S4_fig

S1_table

S2_table

S3_table

Reviewers' comments:

Reviewer #1: I recommend the authors to mention the following findings related to gender, COVID-19 and mental health in the Introduction and discussion, I will review this paper again.

*R= We have reviewed the articles proposed by the reviewer and written brief comments that summarize their vision, as follows:*

*At the same time, during the COVID 19 pandemic, numerous reports were made that indicated increased levels of anxiety, stress, and a greater psychological impact on women.*
